# Comparison of Outcomes of Less Invasive Surfactant Administration in Prematurely Born Infants in the Delivery Suite and the Neonatal Unit

**DOI:** 10.1055/a-2142-9434

**Published:** 2023-08-24

**Authors:** Sandeep Shetty, Donna Tolentino, Anay Kulkarni, Donovan Duffy, Anne Greenough

**Affiliations:** 1 Neonatal Intensive Care Centre, St. George's University Hospitals NHS Foundation Trust, London, United Kingdom; 2 St George's University of London, London, United Kingdom; 3 Department of Women and Children's Health, School of Life Course Sciences, Faculty of Life Sciences and Medicine, King's College London, United Kingdom

**Keywords:** LISA, surfactant administration, admission temperature, duration of mechanical ventilation, length of hospital stay

## Abstract

**Objective** This study aimed to compare outcomes of infants who received less invasive surfactant administration (LISA) in the delivery suite (LISA-DS) with those who received LISA on the neonatal unit (LISA-NNU).

**Study Design** A prospective cohort study was undertaken of all infants who received LISA in a single center. Clinical outcomes included admission temperature, the need for intubation, durations of invasive and noninvasive ventilation, length of hospital stay and the incidences of bronchopulmonary dysplasia (BPD), intraventricular hemorrhage (IVH), retinopathy of prematurity (ROP), and requirement for home oxygen were compared between the two groups as were complications of the procedure.

**Results** The 54 LISA-DS infants had similar gestational ages and birth weights to the 26 LISA-NNU infants ( *p* = 0.732, 0.928, respectively). There were no significant differences between the admission temperatures (median [range]: 36.8 [36–38.7] vs. 36.8°C [36.4–37.7]; *p* = 0.451) or need for intubation in less than 72 hours of birth (28 vs. 23%, *p* = 0.656). The durations of invasive ventilation (median: 2 [0–65] vs. 1 [0–35] days; *p* = 0.188) and noninvasive ventilation (median: 37 [24–81] vs. 37 [3–225] days; *p* = 0.188) and the incidences of BPD ( *p* = 0.818), IVH ( *p* = 0.106), ROP ( *p* = 0.526), and home oxygen requirement ( *p* = 0.764) were similar. The percentage of successful first attempts with LISA (63 vs. 70%, *p* = 0.816) or associated with hypoxia episodes (32 vs. 42%, *p* = 0.194) did not differ significantly by site of administration.

**Conclusion** The outcomes of LISA performed on the DS were similar to those of LISA performed on the NNU.

**Key Points**

Prematurely born infants who received LISA in the DS had comparable clinical outcomes to infants who received LISA on NNU.

No significant differences in admission temperature was noticed in infants who received LISA, in DS versus NNU.

Increasing use of noninvasive ventilation (NIV) has led to the development of a technique, which delivers surfactant without resort to intubation. During less invasive surfactant administration (LISA), surfactant is delivered directly into the lungs via a fine bore catheter inserted into the trachea. 1 The European Consensus Guidelines (2019) on the management of respiratory distress syndrome (RDS) stated that LISA rather than INSURE (INtubation-Surfactant-Extubation) was the preferred mode of surfactant administration for spontaneously breathing preterm babies supported by continuous positive airway pressure. 2 A systematic review of six randomized controlled trials demonstrated that LISA use in infants with RDS was associated with a reduced incidence of bronchopulmonary dysplasia (BPD) and death at 36 weeks and the need for mechanical ventilation; 1 the latter outcome was confirmed in a further systematic review. 3 A survey in 2017 with a 51% response rate, demonstrated that LISA was being used in 48% of European units. 4 That survey, however, did not clarify the location of LISA administration, that is if it was done on the delivery suite (DS) or neonatal unit (NNU). A UK-based survey of all 196 NNUs in 2018 with a 95% response rate, however, demonstrated that only 18 % of NNUs used LISA regularly and only 2% performed LISA in the DS. 5 A subsequent UK-based survey reported in 2020 reported that 56% units would consider LISA on the DS. 6 Furthermore, LISA in the DS has recently been reported to improve clinical outcomes when used in a tertiary NNU in the UK. 7 In a multivariate logistic regression model, of the six independent risk factors identified, the core temperature at the time of neonatal intensive care units (NICUs) admission showed a strong positive correlation with LISA success (odds ratio: 3.56; 95% confidence interval: 1.715–7.394). 8 Lower body temperatures of preterm newborns at admission to NICUs is inversely associated with increased morbidities and mortalities before discharge. 9 10 11 Therefore, maintaining a body temperature of 36.5 to 37.5°C is recommended during resuscitation preterm infants. We have been offering LISA since 2018 both on the NNU and in the DS. 12 Our aim, therefore, was to determine if the outcomes of LISA given to prematurely born infants in the DS particularly admission temperature, were comparable to those in whom LISA was given on the NNU.

## Materials and Methods

All inborn infants born at less than 32 weeks of gestation between July 2018 and July 2022 and who received LISA were included in the study. Infants with major congenital abnormality were excluded from the analysis. The Health Research Authority Toolkit of the National Health System, United Kingdom, confirmed that the study would not need regulatory approval by a research ethics committee.

All LISA procedures were performed by the medical and advanced nurse practitioner team using video laryngoscopy according to the unit's protocol. LISA was delivered via a LISAcath or Surfcath, a thin straight catheter that was passed through the vocal cords and into the trachea. Infants received LISA in the DS if they were transitioned to noninvasive support in the DS and required a fraction of inspired oxygen (FiO _2_ ) of more than 0.3 or had an increased work of breathing with an FiO _2_ requirement less than 0.3. On the NNU, infants had LISA if they were receiving noninvasive support and were less than 72 hours of postnatal age and their FiO _2_ requirement had increased to more than 0.3 or had an increased work of breathing (excluding pneumothorax) or worsening blood gases with a respiratory acidosis (pH < 7.2, PCO _2_ > 8.7 kPa). Infants had oxygen saturation monitoring in the DS and NNU and this guided the inspired oxygen concentration administered in both locations, in addition in the NNU infants also had arterial blood gas monitoring. The dosage of surfactant was aimed to be between 100 and 200 mg/kg and rounded closest to 120 or 240 mg to minimize vial use. Nonpharmacological methods for analgesia such as swaddling, sucking on a dummy or sucrose were used when LISA was performed on the DS or on the NNU. No sedation was given, if LISA was performed on the DS. If the baby remained unsettled when LISA was being undertaken on the NNU, then fentanyl was administered (0.5–1 µg/kg/dose). A loading dose of caffeine (20 mg/kg) was administered after admission to the NNU.

Adverse outcomes compared were the number of LISA attempts, failure of the procedure defined as inability to perform the procedure or need for intubation during the procedure, need for fentanyl and hypoxic episodes defined as desaturation <85% SpO _2_ during the procedure. Other outcomes compared were the admission temperature, need for intubation prior to 24 and 72 hours of postnatal age, the number of surfactant doses and postnatal corticosteroid courses, the durations of invasive and NIV ventilation days and the total length of hospital stay (LOS), the incidences of BPD (oxygen requirement at 36 weeks corrected age), grade 3 or greater intraventricular hemorrhage (IVH), grade 3 or greater retinopathy of prematurity (ROP) requirement for supplementary oxygen at home (home oxygen), and oral injuries such as trauma or bleeding were also compared.

Data were obtained from the electronic documentation recording system, iclip (patient administration system) and standardized electronic neonatal database (Badgernet).

### Sample Size

The mean (standard deviation) of the NNU admission temperature of infants who had not undergone LISA on the DS was 36.8°C (0.42). Analysis of at least 25 infants into each group would allow detection of a difference in the admission temperature of one standard deviation with greater than 90% power at the 5% level of significance.

### Analysis

Differences between the two groups were assessed for statistical significance using the chi-square or Mann–Whitney test as appropriate. IBM SPPS statistical software, V.27 (IBM Corporation, Armonk, NY) as used.

## Results

A total of 80 LISA infants were identified. The 54 LISA-DS infants had similar gestational ages and birth weights to the 26 LISA-NNU infants, ( *p* = 0.732, 0.928, respectively; Table 1 ). LISA was administered in DS at median age of 18 (range: 5–35) minutes and NNU median age of 4 (range: 1–36) hours. A consultant neonatologist as a senior clinician was present in 85% of the LISA-DS group and 73% in the LISA-NNU group ( *p* = 0.32). All infants had FiO _2_ requirement more than 0.3 as the predominant reason for administering LISA. There were no significant differences between the admission temperature ( *p* = 0.451), need for intubation in less than 24 hours ( *p* = 0.107) or less than 72 hours ( *p* = 0.656) from birth, surfactant doses ( *p* = 0.249), postnatal corticosteroid use ( *p* = 0.955), LISA failure episodes ( *p* = 0.489), the durations of invasive ventilation ( *p* = 0.188), or NIV ( *p* = 0.188). The incidences of BPD ( *p* = 0.818), IVH grade 3 or greater ( *p* = 0.106), ROP grade 3 or greater ( *p* = 0.526), and home oxygen requirement ( *p* = 0.764) were similar in the LISA-DS and LISA-NNU infants ( Table 2 ). There were three infants (5.5%) in the LISA-DS group who had temperature below than 36.5°C compared with one (3.8%) in the LISA-NNU group. There were no reported oral injuries in either the LISA-DS or LISA-NNU infants. The percentage of successful first attempts with LISA (63 vs. 70%; *p* = 0.816) or associated with hypoxia episodes (32 vs. 42%; *p* = 0.194) did not differ significantly. Fentanyl was used in the LISA-NNU group (14%) and in none of the NNU-DS group ( *p* < 0.001; Table 2 ).

**Table 1:** Demographic data

	LISA-DS	LISA-NNU	*p* -Value
	( *n* = 54)	( *n* = 26)	
BW (g)	960 (550–1,990)	930 (540–1,810)	0.928
GA (wk)	27.8 (25.0–31.7)	27.9 (24.3–31.7)	0.732
Gender (male)	37 (69%)	14 (54%)	0.201
Antenatal corticosteroids	53 (98%)	25 (96%)	0.593
Senior clinician (consultant) presence	46/54 (85%)	19/26 (73%)	0.32

Abbreviations: BW, birth weight; DS, delivery suite; GA, gestational age; LISA, less invasive surfactant administration; NNU, neonatal unit.

Note: Data displayed as median (range) or *n* (%).

**Table 2:** Outcomes by less invasive surfactant administration status

	LISA-DS ( *n* = 54)	LISA-NNU ( *n* = 26)	*p* -Value
Admission temperature (°C)	36.8 (36–38.7)	36.8 (36.4–37.7)	0.451
Need for intubation < 24 h	9 (17%)	1 (4%)	0.107
Need for intubation < 72 h	15 (28%)	6 (23%)	0.656
Surfactant doses	1 (1–3)	1 (1–3)	0.249
Postnatal corticosteroids	6 (11%)	3 (12%)	0.955
Fentanyl	0 (0%)	13 (24%)	<0.001
LISA failure	1 (2%)	0 (0%)	0.489
LISA success with first attempt	34 (63%)	18 (70%)	0.816
Hypoxia (<85% SpO _2_ )	17 (32%)	12 (46%)	0.194
Duration of invasive ventilation (d)	2 (0–65)	1 (0–35)	0.188
Duration of NIV ventilation (d)	37 (24–81)	37 (3–225)	0.188
Overall LOS (d)	76 (34–176)	69 (24–260)	0.238
BPD	23 (43%)	12 (46%)	0.818
IVH grade 3 or greater	5 (9%)	0 (0%)	0.106
ROP grade 3 or greater	4 (7%)	1 (4%)	0.526
Home oxygen requirement	14 (31%)	9 (35%)	0.764

Abbreviations: BPD, bronchopulmonary dysplasia; DS, delivery suite; IVH, intraventricular hemorrhage; LISA, less invasive surfactant administration; LOS, length of stay; NNU, neonatal unit; ROP, retinopathy of prematurity.

Note: Data displayed as median (range) or *n* (%).

## Discussion

We have demonstrated that prematurely born infants who received LISA in the DS had comparable clinical outcomes to infants who received LISA on NNU. There are advantageous of offering LISA in DS, with earlier respiratory benefits. 13 On the other hand, DS LISA administration could theoretically prolong care in the DS leading to issues such as hypothermia. Importantly, we saw no significant differences in admission temperature between the two groups. Indeed, only three infants (5.5%) in the LISA-DS group had a temperature below than 36.5°C compared with one (3.8%) in the LISA-NNU group. In a retrospective observational study of 5,277 very low birth weight infants, for every 1°C decrease in admission temperature below 36.5°C, there was a 11% increase in developing late-onset sepsis and a 28% increase in the rates of dying. 14 Furthermore, in a retrospective observational study in 29 NICUs in the Canadian Neonatal Network assessing outcomes of 9,833 inborn infants of less than 33 weeks of gestation, the lowest rates of adverse outcomes were associated with admission temperatures ranging from 36.5 to 37.2°C LISA in a nonsedated newborn baby could theoretically increase the risk of trauma and failure of procedure, but we saw no significant differences in oral injury or failure of the technique between the two sites of administration.

Currently, there is no consensus with regard to location of LISA. We had more patients with LISA in the DS than in the NNU. This likely reflects we followed a protocol according to severity of disease and infants who had LISA in the DS had less severe disease when arriving on the NNU, hence were not eligible for LISA. It is important to note that the team had undertaken LISA on the NNU several years before using it in the DS, and this may account for the lack of differences in adverse effects. 12 In a Cochrane review, 15 which included 16 randomized controlled trials comparing surfactant administration via thin catheter (S-TC) with surfactant administration through an endotracheal tube (S-ETT), found the need for intubation within the first 72 hours was 36% in the S-ETT group and 23% in the S-TC group. Those results are comparable to ours where intubation within 24 hours ranged between 4 and 17% and within 72 hours ranged between 23 and 28%.

LISA is not a single technical procedure, but rather a component of a complex care bundle supporting the individual premature baby to adapt to extrauterine life. It is important to prevent hypothermia during the procedure, and it was reassuring to note that there was no significant difference in admission temperatures between the LISA-DS and NNU groups.

In the Nonintubated Surfactant Application (NINSAPP) trial 16 and a meta-analysis, 17 LISA was shown to significantly reduce the incidence of IVH compared with that within the controls. The incidences of IVH in those studies were between 8 and 10.3%. None of those studies, however, had IVH as a primary endpoint. The IVH incidence in our study was 9%.

Nonpharmacological methods for analgesia such as swaddling, sucking on a dummy or sucrose were used when LISA was performed on the DS or on the NNU. A variety of drugs in other studies have been studied for analgesia/sedation during LISA on the NNU; fentanyl, ketamine, and propofol were the most frequently used medications. Studies indicate that these drugs may help to reduce pain scores but can interfere with spontaneous breathing. 18 Indeed, in one study the incidence of desaturation (SpO _2_ < 85%) during LISA was significantly higher in the sedated group (91 vs. 69%, *p* = 0.023) and infants more often needed nasal intermittent mandatory ventilation during the procedure (93 vs. 47%, *p* < 0.001). 18 Fentanyl can cause chest rigidity and interference with spontaneous breathing. Stress and pain in the neonatal period may have long-term negative effects and should be avoided, whenever possible, but drugs used for stress/pain relief also have acute and long-term side effects. 19

## Strengths and Limitations

There are strengths and some limitations to our study. We believe this is the first single-center study that compares LISA outcomes in the DS with those in the NNU. Our sample size was based on the admission temperature as there has been concern that LISA in the DS might increase the incidence of low temperatures. As we report the results of a relatively small sample and we cannot robustly conclude the incidences of IVH and BPD were similar. The baseline demographics of the two groups, however, were not statistically significant, and thus, the lack of significant differences in outcomes is reassuring. The optimal study design would be to randomly assign to infants to LISA in the DS or NNU, but many practitioners prefer to administer surfactant only when the infant has signs of respiratory distress whether this was in the DS or NNU; hence, a randomized study regarding location would not be possible.

## Conclusion

In conclusion, we have demonstrated that the outcomes of LISA given either in the DS or the NNU were similar. Importantly, there were no significant differences in the NNU admissions temperatures according to where the infants had LISA.
